# Screening for ROP

**Published:** 2017-11-11

**Authors:** Anand Vinekar

**Affiliations:** 1 Programme Director: KIDROP, Professor & HoD: Department of Pediatric Retina Narayana Nethralaya Eye Institute, Bangalore, India.

**A low-cost, portable camera developed in India, 3Nethra Neo has made transport, imaging and scaling-up of the KIDROP programme easier.**

**Figure:**
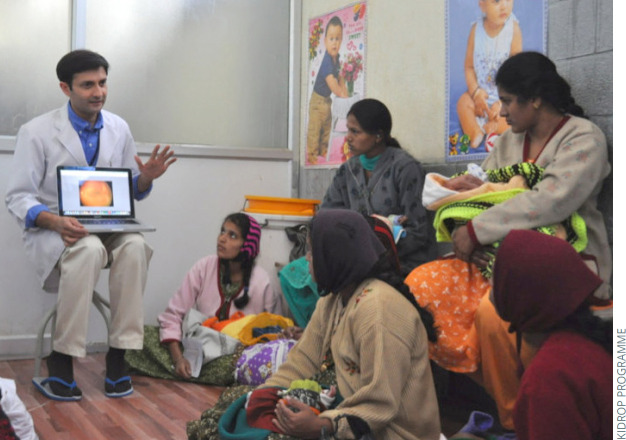
Mothers being shown images of the retinae of their infants during a counseling session. INDIA

Screening is an essential first step in management of retinopathy of prematurity (ROP). This requires training, skill, patience and appropriate equipment. Identification of a child requiring treatment for ROP has a short window period, and a high-risk of poor outcome if the diagnosis is missed.

Although indirect ophthalmoscopic retinal examination is the standard for examining the retinae of infants, imaging based examination and screening is gaining popularity. This article focuses on the use of wide-field imaging as the primary method in ROP screening. This is especially relevant in countries that lack ROP specialists. ‘Wide-field’ in the context of ROP would be 120-degree field of view (or greater), as the disease affects the peripheral retina first. Photographic documentation is a powerful tool in recording medical findings. This also serves as strong medical and legal evidence.

## Can wide-field imaging based ROP screening be used in countries with few ROP specialists?

India with a population of 1.3 billion people, has less than 1,000 trained retinal surgeons, of which less than 150 are currently practicing management of ROP programmes. With a majority of rural areas devoid of such experts, wide-field imaging performed within the neonatal intensive care unit (NICU) by trained and accredited non-physicians could help to bridge the gap.

## Who can take images of infants?

Practically anyone with aptitude can be trained to take ROP-related images of infants. In the Karnataka Internet Assisted Diagnosis of Retinopathy of Prematurity (KIDROP) programme (www.kidrop.org) in India; doctors, ophthalmic imagers, optometrists, nurses and para-medics have been trained to capture retinal images as well as review, record and report them within minutes. They can help them act as the ‘first triage’ in the community, enabling ‘on-the-go’ diagnosis for mothers living in rural areas, even before the ROP specialist reviews these via a tele-medicine platform.^1–3^

## What equipment may be useful in South Asia?

The most commonly used camera worldwide is the RetCam. The ‘shuttle version’ of this device is portable using a four-wheeler and can be moved between several NICUs. The camera is rugged and can withstand the rigors of a tropical climate and rural roads. In the KIDROP programme, 110 NICUs are covered by five cameras, each managed by a separate team. On average, 7000 kilometres of travel are undertaken to reach these centres wherein 1500–2000 imaging sessions are performed every month.^1^,^3^ More recently, a low-cost, portable camera that was developed in India, 3Nethra Neo has made transport, imaging and scaling-up of the programme easier.

## What kind of on-site imaging and documentation can be expected?

Once the device reaches the scheduled centre, it is wheeled into the NICU where the identified infants have their eyes dilated by the nurses. Images can be taken inside the incubator or in an adjoining ‘step-down’ room. A special infant wire speculum keeps the eye open. The camera comes into contact with the cornea with a coupling agent between the two surfaces. Images are captured in the video mode, to reduce movement. Artefacts and the required quadrants are saved as still images. A ROP card is filled out for the mother with the diagnosis and date for next follow-up. Images are used to educate the mother and the treating neonatologist. This reduces follow-up attrition. Findings are recorded and maintained in an onsite register and online. All images are backed up on to a secure online database and are read by the remote expert.^1^,^2^

## How are images reported?

To aid reporting by non-physicians a ‘decision-aiding algorithm’ was developed with three-way triaging code of

- red (requiring treatment or urgent review by the MD),
- orange (disease or immature retina that can be followed) and
- green (mature retina in both eyes).

The algorithm is based on the International Classification of ROP (ICROP). The remote specialist views and reports these images on his or her smart phone. Over 97% sensitivity is possible while reporting on the smart phone. Owing to low internet speeds in some rural areas, the upload time for a single infant's images can range from two to over 15 minutes. The reporting time on an average after upload is four minutes. The time taken to report all ‘severe cases’ of any session that need urgent attention is less than 30 minutes.^3^

## How are imagers trained and accredited?

A KIDROP STAT (Score for Training and Accreditation of Technicians) score has been developed to train and certify imagers. This comprises of three levels (I, II and III) and has a 20 point score, which tests the knowledge, skill, and practise patterns of the imager in their native setting. On an average, training a new imager can take between 30 and 90 working days. This period has been considerably shortened after the introduction of online training.

**Figure:**
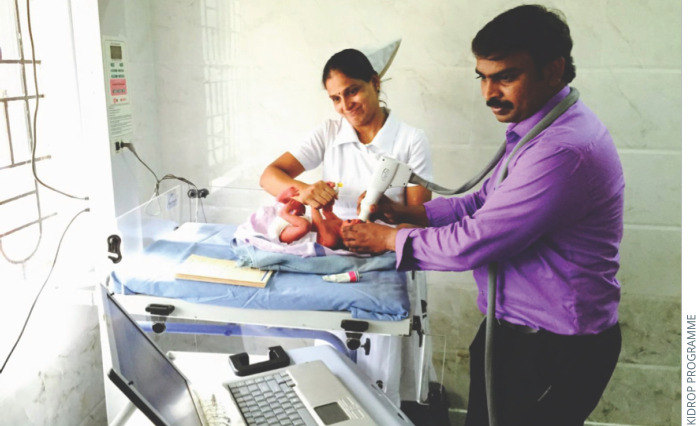
Wide-field imaging being performed by a level III imager in a rural neonatal intensive care unit using the portable RetCam Shuttle. INDIA

## What are other uses of imaging?

Wide-field imaging used for ROP screening has helped diagnose and manage other ‘non-ROP’ conditions in preterm infants. In a study of 1,450 preterm infants, 7.7% had a diagnosis other than ROP, which included conditions as severe as retinoblastoma. Imaging can also be easily performed for anterior and posterior segment screening of full term, healthy infants to provide ‘universal eye screening’. A study of 1,021 term infants imaged within three days of birth showed that 4.7% had an abnormality, 1% of whom required medical or surgical intervention. As more affordable cameras become available, the role of imaging is likely to expand under the national programme to provide universal eye screening at birth.

**Figure:**
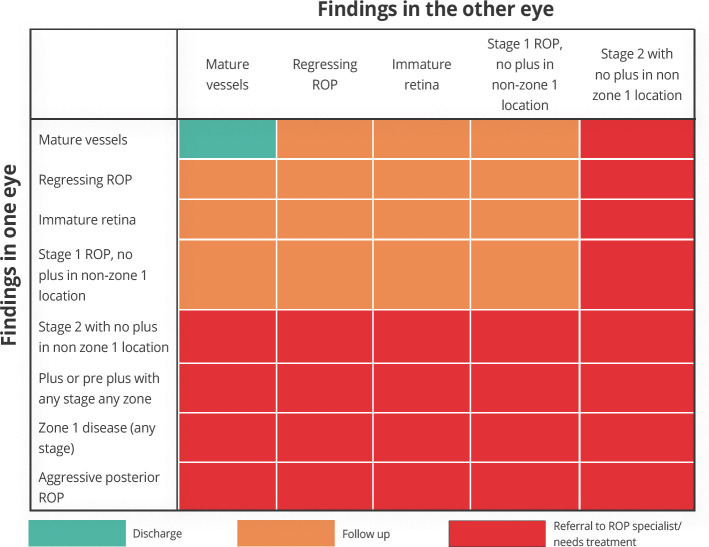


**Figure:**
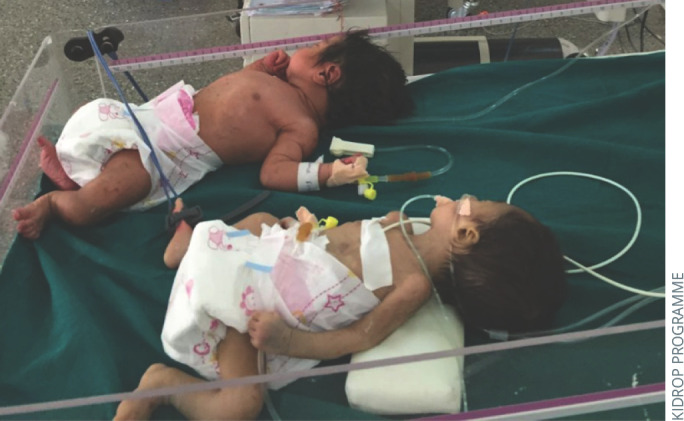
Rural Government owned neonatal intensive care unit which are overburdened with admissions and limited resources. INDIA

## What is the impact of tele-screening?

An impact assessment of scaling up the image based tele-ROP programme in India showed that in the 10 high-risk ROP states, with a population of roughly 680 million, over 35,000 infants would be detected with ROP and over 1,200 need treatment annually. The financial saving in ‘blind-person-years’ (BPY) is estimated at USD 108 million.^4^ Over 650 government owned special new born care units (SNCUs) are already functional in most of the district headquarters in India and many private NICUs also exist. Most of these centres are currently not providing in-house ROP screening. This gap must be met. The United Nations Development Programme (UNDP) report on the tele-imaging programme and the National Health and Medical Research Council (NHMRC, Australia) report based on the Center for Disease Control (CDC) guidelines^5^ on the KIDROP programme both strongly suggest that wide-field imaging is likely to become the new gold standard in ROP screening, and similar models would allow rapid replicability and sustainability in countries like India and others with similar ROP demographics.
